# Prevalence and correlates of neurocognitive impairment among older persons in rural Eastern Uganda

**DOI:** 10.1038/s44400-026-00077-9

**Published:** 2026-04-27

**Authors:** Stephen Ojiambo Wandera, Marleny Nolasco, Shafiq Kawooya, Leah H. Rubin, Noeline Nakasujja, Monica M. Diaz

**Affiliations:** 1 Department of Population Studies, School of Statistics and Planning, College of Business and Management Sciences, Makerere University, Kampala, Uganda; 2 Department of Neurology, University of North Carolina at Chapel Hill School of Medicine, Chapel Hill, NC USA; 3 Departments of Neurology, Psychiatry and Behavioral Sciences, and Molecular and Cellular Pathobiology, Johns Hopkins School of Medicine, Baltimore, MD, USA, Department of Epidemiology, Bloomberg School of Public Health, Baltimore, MD USA; 4 Department of Psychiatry, School of Medicine, College of Health Sciences, Makerere University, Kampala, Uganda

**Keywords:** Diseases, Health care, Medical research, Neurology, Risk factors

## Abstract

The population of adults aged 60 years and older in sub-Saharan Africa is projected to reach 670 million by 2030. This study aimed to determine the prevalence and risk factors for dementia among older adults in rural eastern Uganda. We conducted a cross-sectional study from December 2023 to September 2024 in eastern Uganda. Cognitive function was assessed using the Identification and Intervention for Dementia in Elderly Africans and a functional assessment. Sociodemographic, medical, social characteristics were captured. Multivariable logistic regression identified factors associated with neurocognitive impairment. Among 598 older adults (mean age 70.0 ± 9.0 years; 60.6% female), 39% had no formal education. We found 20.6% had neurocognitive impairment and 12% had dementia. Older age or being underweight were significant risk factors for neurocognitive impairment. There is a high burden of neurocognitive impairment among older adults in rural Uganda. Addressing modifiable risk factors through public health programs is essential for dementia prevention.

## Introduction

Globally, about 55 million people live with dementia with approximately 10 million new cases of dementia each year^1,2^. The burden of dementia is particularly significant in LMICs, where nearly 60% of people with dementia reside, and this proportion is projected to rise to 70% by 2050^2^. In Uganda, people over age 60 comprise 5% of the population^3^, however the Ugandan population is aging with reductions in mortality from communicable diseases and rising dementia rates worldwide. These shifts highlight the need to identify and address modifiable risk factors of dementia, particularly in rural, underserved areas with limited healthcare access in sub-Saharan Africa (SSA).

Rural eastern Uganda has several poor health indicators driven by limited healthcare access, high poverty rates, and a significant burden of disease. For example, mortality rates of children under 5 years of age are elevated compared to national averages^4^. Infectious diseases, such as tuberculosis and malaria, also remain prevalent, and recently, blackwater fever, a severe complication of malaria, has been rising^4,5^. Moreover, eastern Uganda is one of the poorest and most rural regions in Uganda, with one of the lowest socio-economic and health indicators in the country^6^. According to the Uganda Population and Housing Census 2022^4^, both Busia and Namayingo districts have poor health and demographic indicators with high poverty rates, with limited access to clean water, adequate nutrition, and healthcare^4^. Moreover, healthcare infrastructure remains inadequate, with only 60% of rural residents living within 5 kilometers of a health facility^7^. These factors contribute significantly to the care of older adults with comorbid conditions of aging, including neurocognitive impairment and/or dementia.

The 2024 Lancet Commission identified fourteen modifiable risk factors that, if modified, may reduce dementia incidence by up to 40%^4,5^. Based on one systematic review and meta-analysis, dementia risk factors reported in the literature in SSA include lower education levels, vascular risk factors, and socioeconomic disparities^8–10^. Older age remains the most significant independent risk factor, as well as family history of dementia, poor social engagement, hypertension, and undernutrition, which were risk factors for dementia in SSA^8,11,12^. Moreover, mental health conditions, such as depression and anxiety, are increasingly recognized as risk factors for cognitive decline^8^.

Despite these known modifiable dementia risk factors in SSA that could significantly reduce the incidence of dementia, studies on dementia risk factors in the region, including rural Uganda, are generally lacking. Understanding the influence of these factors on dementia prevalence is essential for developing culturally appropriate dementia prevention and intervention strategies for rural SSA. In this study, we sought to determine the prevalence and risk factors associated with dementia among older adults residing in two districts of rural eastern Uganda.

## Results

### Demographic characteristics

We enrolled 602 older adults meeting the inclusion criteria and interviewed 602 informants in the household. We excluded 4 participants with incomplete data. We therefore analyzed data from 598 older adults in our study with a mean+/− standard deviation (SD) age of 70.0+/−9.0 years, 60.7% females **(**Table 1**)**. Nearly 40% of all older adults had no formal education. The most common source of livelihood was farming (66%), and more than 40% were Catholics, and nearly all had birthed or fathered a child (98%). The majority (72%) were not involved in remunerated work (data not shown).

**Table 1:** Frequency of demographic and clinical characteristics of participants (*N* = 598)

	Frequency (*n*)	Percent (%)
**Demographics**		
**District**		
Busia	323	54
Namayingo	275	46
**Sex**		
Female	363	60.7
Male	235	39.3
**Age group**		
60–69	334	55.9
70–79	163	27.3
80+	101	16.9
**Education level**		
No education	232	38.8
Primary or higher	366	61.2
**Religion**		
Catholic	243	40.6
Anglican	168	28.1
Pentecostal or others*	187	31.3
**Marital status**		
Formerly married	354	59.2
Currently married	244	40.8
**Clinical Characteristics**		
**Depression based on PHQ-9**		
None or minor depression	269	45
Moderate to severe depression	329	55
**Blood Pressure**		
Normotensive	471	78.8
Hypertensive	127	21.2
**Diabetes**		
No hyperglycemia	551	92.1
Hyperglycemia	47	7.9
**Body Mass Index** (kg/m^2^)		
Malnutrition (BMI < 18.5)	153	25.6
Normal BMI	327	54.7
Overweight or obese	118	19.7
**Alcohol problems using cage scale**		
No	427	71.4
Yes	171	28.6
**Ever or currently smoking**		
No	527	88.1
Yes	71	11.9
**Living alone and socially isolated**		
No	292	48.8
Yes	306	51.2
**Source of energy for lighting**		
Electricity or solar	306	51.2
Tadooba or paraffin lamp	166	27.8
Firewood or others	126	21.1
**Dementia based on IDEA Tool**		
No dementia	475	79.4
Probable dementia	123	20.6
**MCI based on IDEA** + **IDEA-IADL tool**		
No	547	91.5
Yes	51	8.5
**Dementia based on IDEA** + **IDEA-IADL tool**		
No	526	88
Yes	72	12
**Total**	**598**	**100**

*BMI* Body Mass Index, *IDEA* Identification and intervention for dementia in elderly Africans, *MCI* mild cognitive impairment, *PHQ-9* Patient Health Questionnaire-9.

*Other religions were Muslim (*n* = 51), New Apostolic Church (*n* = 4), Isa Masiya (*n* = 3), Legio Maria (a Catholic cult based in Kenya, *n* = 1.

### Medical and behavioral characteristics

We found that only 19.7% were obese, nearly 7.9% had hyperglycemia, 21% had hypertension. About 12% were current or former smokers, and nearly 30% had a possible alcohol use disorder. We found that about half were living alone and socially isolated, and 55% had moderate to severe depression based on the PHQ-9 **(**Table 1**)**.

### Neurocognitive health outcomes

We found that 20.6% of all older adults had possible neurocognitive impairment based on the IDEA tool alone. While 8.5% had MCI and 12% had dementia based on both the IDEA cognitive screening tool and the IDEA-IADL tool **(**Table 1**)**.

There were statistically significant differences between risk factors for older adults who were cognitively healthy compared with those with possible neurocognitive impairment. Those from Namayingo, people 80 years and older, females, those with no formal education, having moderate to severe depression on the PHQ-9, being underweight, current or former smokers, living alone and socially isolated, or using a paraffin lamp as the primary source of lighting had a significantly higher frequency of neurocognitive impairment. Those who were Pentecostal or other religions (including Muslim, The religious affiliation categories included: Muslim (*n* = 51), New Apostolic Church, Isa Masiya (Christian cult in East Africa), Legio Maria (a Catholic cult based in Kenya), those who were currently married, had the lowest frequency of possible neurocognitive impairment **(**Table 2**)**.

**Table 2:** Risk factors for possible neurocognitive disorder based on IDEA cognitive screening tool (*N* = 598)

	Neurocognitively healthy	Possible NCD	*P*-Value
	%	%	
**District**			**0.02**
Busia	83.0	17.0	
Namayingo	75.3	24.7	
**Sex**			**<0.001**
Female	73.8	26.2	
Male	88.1	11.9	
**Age group**			**<0.001**
60–69	89.8	10.2	
70–79	75.5	24.5	
80+	51.5	48.5	
**Education level**			**<0.001**
No education	64.7	35.3	
Primary or higher	88.8	11.2	
**Religion**			**0.025**
Catholic	74.9	25.1	
Anglican	79.2	20.8	
Pentecostal or others*	85.6	14.4	
**Marital status**			**<0.001**
Formerly married	71.8	28.2	
Currently married	90.6	9.4	
**Depression based on PHQ-9**			**<0.001**
None or minor depression	87.0	13.0	
Moderate to severe depression	73.3	26.7	
**Hypertension**			0.60
Normal BP	79.0	21.0	
Hypertensive	81.1	18.0	
**Possible diabetes** (based on random blood glucose level)			0.90
No diabetes	79.5	20.5	
Possible diabetes	78.7	21.3	
**Body Mass Index**			**0.004**
Normal	79.8	20.2	
Underweight	71.9	28.1	
Overweight or obese	88.1	11.9	
**Alcohol problems using the Cage scale**			0.63
No	78.9	21.1	
Yes	80.7	19.3	
**Ever or currently smoking**			**0.001**
No	81.4	18.6	
Yes	64.8	35.2	
**Living alone and socially isolated**			**<0.001**
No	89.7	10.3	
Yes	69.6	30.4	
**Source of Energy for Lighting**			**<0.001**
Electricity or Ssolar	83.7	16.3	
Paraffin lamp	68.7	31.3	
Firewood or others	83.3	16.7	

*PHQ-9* Patient Health Questionnaire-9.

**Other religions were Muslim (*n* = 51), New Apostolic Church (*n* = 4), Isa Masiya (*n* = 3), Legio Maria (a Catholic cult based in Kenya, *n* = 1.

**Fig. 1:**
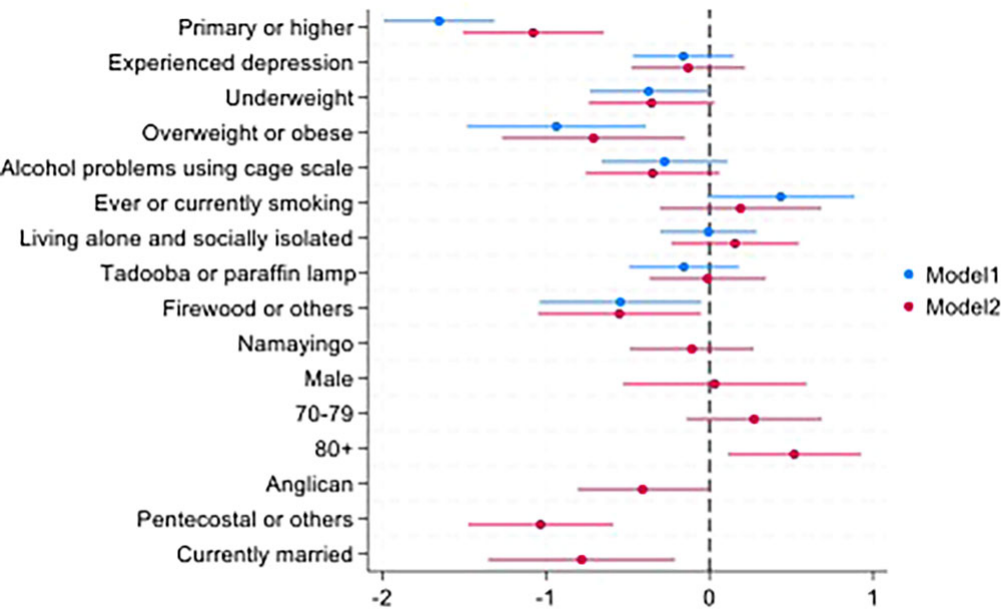
Coefficient plot for risk factors for possible neurocognitive disorder based on IDEA Cognitive Screening Tool (*N* = 598)

In multivariable regression analyses, we found that completion of primary school or higher, being overweight or obese, using firewood or other fuels, being Pentecostal or other religion, or being currently married were protective factors against probable neurocognitive impairment. Age 80 years or older was a significant risk factor for probable neurocognitive impairment. These protective factors were still statistically significant after removing the socio-demographic covariates (Table 3, Fig. 1**)**.

**Table 3:** Multivariable logistic regression results for risk factors for probable dementia and dementia

	Model 1: Risk factors for dementia only	95% Confidence Intervals (CI)	Model 2: Sociodemographic and dementia risk factors	95% CI
Variables				
Education level				
No education	1		1	
Primary school or higher	0.19^***^	[0.14–0.27]	0.34^***^	[0.22–0.52]
Experienced depression	0.85	[0.63–1.16]	0.88	[0.62–1.24]
**BMI**				
Normal	1		1	
Underweight	0.69^*^	[0.48–0.99]	0.70	[0.48–1.03]
Overweight or obese	0.39^***^	[0.23–0.68]	0.49^*^	[0.28–0.86]
Alcohol problems using the Cage scale	0.76	[0.52–1.12]	0.71	[0.47–1.06]
Ever or currently smoking	1.55	[0.99–2.43]	1.21	[0.74–1.98]
Living alone and socially isolated	0.99	[0.74–1.34]	1.17	[0.79–1.73]
**Source of energy**				
Electricity or solar	1		1	
Paraffin lamp	0.86	[0.61–1.20]	0.99	[0.69–1.41]
Firewood or others	0.58^*^	[0.35–0.95]	0.58^*^	[0.35–0.95]
**District**				
Busia			1	
Namayingo			0.90	[0.61–1.31]
**Sex**				
Female			1	
Male			1.03	[0.59–1.81]
**Age group**				
60–69			1	
70–79			1.31	[0.87–1.99]
80+			1.68^*^	[1.12–2.52]
**Religion**				
Catholic			1	
Anglican			0.66^*^	[0.45–0.99]
Pentecostal or others*			0.36^***^	[0.23–0.56]
**Marital status**				
Formerly married			1	
Currently married			0.46^**^	[0.26–0.81]

Exponentiated coefficients; 95% confidence intervals in brackets; ^*^p < 0.05, ^**^p < 0.01, ^***^p < 0.001.

*Other religions were Muslim (*n* = 51), New Apostolic Church (*n* = 4), Isa Masiya (*n* = 3), Legio Maria (a Catholic cult based in Kenya, *n* = 1.

## Discussion

This study aimed to estimate the prevalence and the correlates of dementia among older people in rural eastern Uganda. We present the first study on neurocognitive impairment and dementia prevalence in a rural eastern Ugandan population, and one of the only indoor-to-door prevalence studies among community-dwelling older adults in Uganda using a regionally validated cognitive screening tool for illiteracy or low literacy levels. The prevalence of possible or probable dementia was 12%, a finding higher than what has been reported from an earlier systematic review of dementia in Africa^13^. Though other studies conducted in Uganda reported a prevalence of 20%^14^, this difference could be accounted for by the instruments used that may not have assessed how functional impairment accompanies dementia.

Our study identified several key protective factors that may lower the risk of neurocognitive impairment, including higher formal education, higher BMI, and being currently married, after controlling for covariates that were statistically significant in the bivariate comparison. We did not find that known risk factors, such as hypertension, hyperglycemia, obesity, social isolation, or depression, were statistically significant in the multivariable regression analyses. However, we found that important risk factors, such as female sex, depression, being underweight, and currently or formerly smoking, were associated with significantly higher frequency of neurocognitive impairment.

Our results suggest that having a normal weight or being obese, compared with being underweight, could be protective of dementia. According to the 2024 Lancet Commission on dementia, obesity is a modifiable risk factor for dementia, which, when eliminated, could lead to a 1% reduction in cases of dementia^15^. In the United States, however, longitudinal associations indicated that an obese BMI was associated with a less steep decline in motor function in men with HIV, whereas in HIV-negative men, obesity was associated with a greater decline in motor function, learning, and memory. WC, or central obesity, showed similar patterns of associations^16^. Other data suggest that inadequate nutrition can influence the progression of Alzheimer’s disease^17^. In older adults, malnutrition often reflects a trajectory of declining health and reduced quality of life, characterized by inadequate dietary intake, muscle wasting, poor appetite, and unintentional weight loss, and is often driven by social and physical factors such as loss, dependency, loneliness, and chronic illness^18^.

In sub-Saharan Africa, prevalence estimates of malnutrition among older adults range from 6% to 48%^19^, and pooled estimates show rates of undernutrition as high as 18%^20^. In some low socio-economic settings, prevalence may reach up to 28.4%, with the highest rates often reported in rural and economically disadvantaged communities^21^. In this context, a higher body mass index in this setting could indicate better nutrition and greater resilience among older adults. The doubling of overweight and obesity prevalence in Ugandan men and women from 2011 to 2022 further highlights changing nutritional patterns that may influence dementia risk. These findings underscore the importance of considering both overnutrition and undernutrition for dementia risk, particularly in regions where malnutrition remains prevalent among older adults.

Social engagement is a critical determinant of cognitive health, yet older adults in Uganda often experience reduced social contact due to widowhood and family migration patterns^22,23^. Loneliness and social isolation have been linked to dementia in various studies. Loneliness can be categorized as emotional loneliness, which involves a lack of an attachment figure and feelings of isolation even when not alone, and social loneliness, which refers to the absence of a social network or community belonging^24^. For instance, a meta-analysis involving 51 longitudinal cohort studies and 102,035 participants aged 50 and older found that high social contact was associated with better cognitive function^25^. Some studies suggest the relationship may be mediated by depressive symptoms, which account for approximately 75% of this connection^12^. Another UK-based 28-year follow-up study of 10,308 people showed that frequent social contact at age 60 reduced dementia risk over 15 years, independent of socioeconomic and lifestyle factors^26^. Individuals with limited social networks, low frequency of social contact, or inadequate social support are at higher risk for cognitive decline and dementia^27^. One US-based study found that loneliness was associated with poorer cognitive performance in executive function and processing speed among older people with HIV^28^. In Uganda, where traditional family structures are increasingly disrupted, promoting social engagement could serve as a protective factor against dementia. Our study found that being currently married and being Pentecostal were each protective factors lowering risk of neurocognitive impairment, but living alone and being socially isolated were not statistically significant, demonstrating that strong bonds, such as those in marriage, help lower neurocognitive risk in this population.

Religious affiliation is known to provide regular social interaction, which leads to cognitive stimulation, which has been shown to be associated with better cognitive function in older adults. Religious participation can also strengthen social support networks and foster a sense of belonging and purpose, which may be factors protecting against life stressors and promote emotional well-being, which may protect against cognitive impairment. Regular involvement in faith activities also offers structured routines and complex mental engagement that can build cognitive reserve over time. Pentecostals may have higher religiosity and more frequent engagement in communal worship compared with other religions, which may influence the relationship between religion and cognition seen in our study.

Although depression was not a significant risk factor in multivariable regression analyses, we found that 26.7% of those with neurocognitive impairment had moderate to severe depression (compared with 13.1% of those without neurocognitive impairment), highlighting that depression may be a significant risk factor. Recently, global research has identified depression as a potential risk factor for dementia, with substantial evidence linking late-life depressive symptoms to increased dementia risk. A meta-analysis of 32 studies, encompassing 62,598 participants, found that depression nearly doubles the risk of dementia over follow-up periods ranging from 2 to 17 years^29^. A 14-year longitudinal study of 4,922 older men also found that depression increased the incidence of dementia by 1.5 times^30^. However, this relationship was strongest among those who developed dementia within five years of experiencing depression, suggesting that depression may be an early symptom of dementia rather than solely a risk factor. In Uganda, the impact of depression on dementia risk is of particular concern, given the high prevalence of underdiagnosed and undertreated mental health conditions^31^.

Another known risk factor for neurocognitive impairment found in our study was having no formal education compared with primary school or higher. Lower levels of formal education represent a significant and modifiable risk factor for neurocognitive impairment. Numerous studies, including hospital-based surveys of older Ugandan adults, have demonstrated that having no formal education is strongly associated with greater odds of cognitive impairment and dementia. One study in a tertiary care hospital reported that lower education was significantly linked to severe neurocognitive impairment among adults aged 60 years and older^32^. Community surveys also highlight this relationship. For example, in a cohort of older Ugandans, individuals with any formal schooling were about 46% less likely to be at risk of dementia compared to those with no education^33^. Moreover, a systematic review across sub-Saharan Africa showed that low educational attainment is among the most prominent modifiable risk factors for incident dementia in the region^8^. The concept of “cognitive reserve” is thought to protect against age-related neurodegeneration, delaying the onset of clinical decline. In Uganda, where formal schooling may be inaccessible in rural areas and particularly among older adults^6^, a lack of formal education contributes to low cognitive reserve, increasing risk of neurocognitive impairments. These findings emphasize the potential of educational interventions, such as late-life or adult-education programs, which have been demonstrated in other studies to be protective against neurocognitive impairments^34^.

Our study also found that using firewood, compared with paraffin lamp or electricity, was a protective factor for neurocognitive impairment. Exposure to household and ambient air pollution is known to be a risk factor for dementia. For example, studies in India, China, and Mexico have consistently found that using polluting cooking fuels is linked with poorer cognitive function and greater cognitive decline^35,36^. Firewood use may be a proxy for more active lifestyles, such as farming or fishing, which may be protective against cognitive impairment. Paraffin lamps may be a source of indoor air pollution (particulate matter 2.5), which is known to be a risk factor for dementia. For example, one study in rural Uganda reported that use of open-wick kerosene lamps generated about twice as much PM2.5 and nearly five times as much black carbon in living rooms compared with cleaner options like solar lighting, even when controlling for household income^37^. Moreover, there may be variables that are highly associated with firewood use that were not captured in this study.

Despite these findings, our study has some strengths and limitations. First, our study was a cross-sectional and door-to-door population-based study, limiting applicability to clinic or hospitalized populations. Second, our primary outcomes, neurocognitive impairment or dementia, were based on brief cognitive screening tools and not on a complete battery of neuropsychological tests. However, the tool utilized had been validated for low literacy and illiterate populations from neighboring Tanzania, strengthening its applicability to our population, but it has not been validated in rural eastern Uganda. Third, our study was limited to two rural districts of eastern Uganda, limiting generalizability to other regions of Uganda or other countries. Next, the CAGE questionnaire does not capture overall alcohol consumption patterns, and future studies may use a more detailed questionnaire. Moreover, although we adjusted for risk factors significant in the bivariate comparisons, there may have been unmeasured covariates, such as adverse social determinants of health, that were unmeasured.

This was the first large cross-sectional door-to-door population-based study of older community-dwelling adults in two rural eastern Uganda districts. Second, our study demonstrated important protective factors, including being currently married, being underweight, and having a higher education, as factors that reduced dementia risk. These factors are important because they highlight the need to improve the nutrition of older adults in the region in order to prevent long-term neurodegenerative consequences. Other factors such as implementing a late-life education program, may help mitigate the onset of cognitive decline. Cognitive stimulation therapy (CST), a structured psychosocial group intervention for people with dementia, has been adapted and piloted in sub-Saharan African settings, and has been followed by significant improvements in cognition^38^. Lastly, forming strong emotional bonds, such as through a healthy marriage, may also help prevent the onset of cognitive decline. Future studies would propose piloting interventions in the community to address these factors and mitigate cognitive decline in these rural regions of sub-Saharan Africa.

## Methods

### Study design and setting

We conducted a prospective, cross-sectional, door-to-door assessment/survey using validated questionnaires. between December 2023 and September 2024.

### Inclusion and exclusion criteria

We included those households in which at least one adult age 60 years or older was available at the time of the home visit, could confirm that he or she lived in the selected household for at least 6 months, and had the ability to provide informed consent to participate in the study (if the person has capacity to consent, or if not able to consent then consent was obtained from a caregiver in the household). In addition, the caregiver needed to be present at the time of the interview with the older adult.

We excluded households in which no eligible household member could be interviewed due to a physical disability limiting ability to participate in cognitive testing (i.e., hearing or visual impairment) or other mental health condition, or those taking medications that may affect cognitive testing performance in the past 7 days (i.e, opiate pain medication). Also, we excluded those whose caregivers were not present and could not be traced on the appointment.

### Sampling strategy

The population of adults aged 60 years and older was 28% according to the 2014 Uganda Population and Housing Census (UPHC), the last census reported in Uganda prior to the beginning of this study^6^. To define older persons, we used the United Nations’ definition of older age (age 60 years and older)^39^.

In each household, a dyad of the older person and a caregiver was interviewed. The Kish’s formula was applied to generate a required sample size of older persons using the prevalence of cognitive disorder estimated at 20% from a cross-sectional study of people living with HIV in rural southwestern Uganda to estimate the expected proportion of dementia in our study^40^.

A multi-stage stratified cluster sampling design was applied using a sampling framework of households with older persons (adults aged 60 years and older) in rural eastern Uganda. We used the sampling framework from the 2014 Uganda Population and Housing Census (UPHC)^6^. We could not access the recent 2024 UPHC sampling frame since it was not yet publicly available.

First, two districts (Busia and Namayingo) were randomly selected from the 46 districts in eastern Uganda using simple random sampling. Second, using computerized random numbers for each sub-county, two sub-counties were selected from each of the two districts. Third, one parish was then selected randomly using computerized random numbers from each sub-county. Fourth, six enumeration areas were selected using simple random sampling. Finally, from each village, in consultation with local leaders, a sampling frame of households with older persons was constructed. Systematic sampling was then applied to select 30 households from each of the 24 villages for the study.

### Outcome variables: dementia screening

To screen for dementia, we used the Identification and Intervention for Dementia in Elderly Africans (IDEA) cognitive screening tool^41^. The IDEA tool is a brief cognitive screening tool validated in rural Tanzania for older adults with low literacy and has demonstrated high diagnostic accuracy for dementia. The tool has been validated in other sub-Saharan African populations, including rural community samples of older adults in Tanzania and Nigeria, with normative values established among these populations^41,42^. The IDEA is scored from 0 to 15, including assessments of abstraction, orientation, long-term memory, categorical verbal fluency, visuospatial construction, and verbal delayed recall. The tool does not require the ability to read, write, draw, or calculate. An external validation study in Tanzania established the following cut-off scores, which we applied for our study: 0–7 “probable dementia”; 8–9 ‘possible dementia”; and 10–15 “no dementia”^41^.

### Functional assessment

All older adult participants underwent a functional impairment screen using the IDEA-Independent Activities of Daily Living (IDEA-IADL) questionnaire^43^. This is a screen comprised of 11 questions scored on a 4-point scale: “cannot do this” (0 points); “can do this with much assistance” (1 point); “can do this with a small amount of help or assistance” (2 points); and “can do this with no difficulty, no help needed” (3 points). The scale was developed specifically for use in a rural Tanzanian setting based on consensus of the types of IADLs an older person might be expected to perform in a rural African setting^43^.

### Neurocognitive health

Each participant was assigned to one of two groups in two separate statistical analyses, one analysis applying the IDEA tool only and another analysis applying the IDEA and the IDEA-IADL tool, denoting their neurocognitive health based on the DSM-V criteria for diagnosing dementia^44^.

- Cognitively normal: Participants who did not report neurocognitive concerns and performed within normal range on the IDEA tool and demonstrated no functional compromise on the IDEA-IADL.
- Neurocognitive impairment: Participants scored in the impaired range based on the IDEA tool alone.
- Mild Cognitive Impairment: (MCI): Participants scored in the impaired range based on the IDEA tool but scored in the normal range on the IDEA-IADL.
- Dementia: Participants with dementia were those who performed in the impaired range on the IDEA and demonstrated functional compromise on the IDEA-IADL.

### Explanatory variables

Using structured questionnaires and trained study personnel, we collected four types of explanatory variables: (1) demographic; (2) home environment (3) social and environmental variables; and (4) medical, behavioral, cognitive, and functional variables pertinent to brain health and neurocognitive impairment risk^15^. We included religious affiliation as a social variable given its relationship with social engagement^45^. The religious affiliation categories included: Catholics, Anglicans, Pentecostals, Muslims, or Others, with an option for writing in the religious affiliation. Pertinent medical history variables included self-reported medical history guided by conditions that are known risk factors for neurocognitive impairment throughout the life course, specifically traumatic brain injury, hypertension, obesity, smoking, depression, and diabetes^15^.

We also measured the following during each assessment of the older adult: blood pressure, weight, and height for body mass index. A trained nurse collected blood pressure readings from each participant. Blood pressure was measured in each ar,m and the mean of both blood pressure readings was calculated. Hypertension was defined as SBP 140 mm Hg or higher and DBP 90 mm Hg or higher^46^. Hyperglycemia was assessed using a random blood glucose test performed during the assessment and defined as random blood glucose > =200 mg/dL.

We collected behavioral and mental health history data pertaining to key behavioral risk factors for neurocognitive impairment, namely smoking and alcohol consumption. Possible alcohol use disorder was measured using the CAGE assessment for alcohol use disorder risk^47^, previously applied in Uganda^48,49^. We asked participants if they currently or formerly smoke(d) cigarettes and the quantity. We measured social isolation using the Social Isolation Scale, comprised of five indicators: marital status, family members for support, monthly contact with friends or family members, participation in organizations, and religious groups; higher scores were indicative of greater social isolation.

Depression screening was completed using the 9-item Patient Health Questionnaire (PHQ-9)^50^, which has been previously applied in Uganda^51,52^. A score of 10 or greater was used to define moderate to severe depression on the PHQ-9^50^. Loneliness was assessed using the UCLA Loneliness Scale – Short Form^53^, previously validated in Zimbabwe^54^.

### Ethical considerations

Ethical approval was obtained from the local Institutional Review Board (TASO Research Ethics Committee (TASO-2022-179*)* and the Uganda National Council of Science and Technology (HS2693ES).

Voluntary informed consent was obtained from all study participants (both the older adult and the caregiver in each household). In the case that the participant was deemed by the research nurse to not have the capacity to provide informed consent, the caregiver in the household provided informed consent.

### Statistical analysis

All survey data was entered into RedCap hosted at Makerere University, College of Health Sciences. Redcap is a secure data collection platform with no collection of identifying information. Data analysis was done using STATA version 19.

For all descriptive statistics, we reported absolute and relative frequencies for categorical variables (e.g., level of cognitive impairment, history of diabetes, etc.), and mean and standard deviations (if normally distributed) or median and interquartile range (if not normally distributed) for continuous variables (e.g., body mass index using calculated BMI, etc.).

Cross-tabulations were used to investigate associations between dementia (outcome variable) and selected explanatory variables. Pearson’s chi-squared (χ2) tests were used to examine the differences between dementia and the explanatory variables. The level of statistical significance using *p*-values was set at *p* < 0.05.

Multivariable logistic regression analyses were used to examine the association between neurocognitive impairment and explanatory variables whose *p*-values were less than 0.05 during the chi-square tests. We used a stepwise regression for multivariable analysis, including all relevant covariates. We controlled for all key dementia risk factors and socio-demographic variables, including district, age group, sex, education, religion, marital status, depression, BMI category, hyperglycemia status, hypertension status, suspected alcohol use disorder, smoking, loneliness, and source of energy for lighting. Results are presented in the form of Odds Ratios (OR) reporting 95% confidence intervals. The level of statistical significance using *p*-values was set at p < 0.05. All analyses were performed in STATA version 19.

## Data Availability

The datasets generated and/or analyzed during the current study are not publicly available due to needing to request from the corresponding author, but are available from the corresponding author on reasonable request.
